# Nuciferine Ameliorates Inflammatory Responses by Inhibiting the TLR4-Mediated Pathway in Lipopolysaccharide-Induced Acute Lung Injury

**DOI:** 10.3389/fphar.2017.00939

**Published:** 2017-12-21

**Authors:** Haichong Wu, Yaping Yang, Shuai Guo, Jing Yang, Kangfeng Jiang, Gan Zhao, Changwei Qiu, Ganzhen Deng

**Affiliations:** Department of Clinical Veterinary Medicine, College of Veterinary Medicine, Huazhong Agricultural University, Wuhan, China

**Keywords:** nuciferine, anti-inflammation, acute lung injury, toll-like receptor 4, nuclear factor-κB

## Abstract

Acute lung injury (ALI) is a complex syndrome with sepsis occurring in critical patients, who usually lack effective therapy. Nuciferine is a primary bioactive component extracted from the *lotus leaf*, and it displays extensive pharmacological functions, including anti-cancer, anti-inflammatory, and antioxidant properties. Nevertheless, the effects of nuciferine on lipopolysaccharide (LPS)-stimulated ALI in mice has not been investigated. ALI of mice stimulated by LPS was used to determine the anti-inflammatory function of nuciferine. The molecular mechanism of nuciferine was performed on RAW264.7 macrophage cells. The results of pathological section, myeloperoxidase activity and lung wet/dry ratio showed that nuciferine alleviated LPS-induced lung injury (*p* < 0.05). qRT-PCR and ELISA experiments suggested that nuciferine inhibited TNF-α, IL-6, and IL-1β secretion in tissues and RAW264.7 cells but increased IL-10 secretion (*p* < 0.05). Molecular studies showed that TLR4 expression and nuclear factor (NF)-κB activation were both inhibited by nuciferine treatment (*p* < 0.05). To further investigate the anti-inflammatory mechanism of nuciferine, TLR4 was knocked down. When TLR4 was silenced, LPS induced the production of IL-1β, and TNF-α was markedly decreased by TLR4-siRNA and nuciferine treatment in LPS-induced RAW264.7 cells (*p* < 0.05). These results suggested that nuciferine had the ability to protect against LPS-stimulated ALI. Thus, nuciferine may be a potential drug for treating LPS-induced pulmonary inflammation.

## Introduction

Acute lung injury (ALI) is a complex syndrome of sepsis in critical patients, which is closely related to high morbidity and mortality (Abdelmageed et al., 2015). ALI’s characteristics are hypoxemia, alveolar capillary membrane disruption, and pulmonary edema (Wu et al., 2016a). In addition, the acute respiratory distress syndrome is a more severe form of ALI, and the mortality rate remains as high as approximately 40% (Huang et al., 2016).

Lipopolysaccharide (LPS), an ingredient of endotoxin in the outer membrane of Gram-negative bacteria, has been widely used to induce ALI in mice (Sun et al., 2010; Zhao et al., 2014; Chao et al., 2017). LPS has great biological activity and enters the bloodstream, leading to serious inflammatory responses (Wu et al., 2016b). It is well-known that the pro-inflammatory mediator secretions, such as IL-1β, IL-6, and TNF-α, will increase sharply (Wang et al., 2011). These excessive expressions of pro-inflammatory cytokines contribute to lung edema and alveolar hemorrhage (Zhao et al., 2014). Moreover, these cytokines are activated by the NF-κB signaling pathway (Cho et al., 2014). TLR4, an important member of the toll like receptor (TLR) family, is needed for the response of LPS and participates in host resistance against Gram-negative bacteria (Wu et al., 2012). An increasing body of evidence has suggested that TLR4 is stimulated by LPS and can activate the NF-κB signaling pathway (Cho et al., 2014; Li et al., 2016; Wang et al., 2017). Thus, this approach may represent a useful strategy for treatment of ALI by interfering with the TLR4 activated signaling pathway.

**FIGURE 1:**
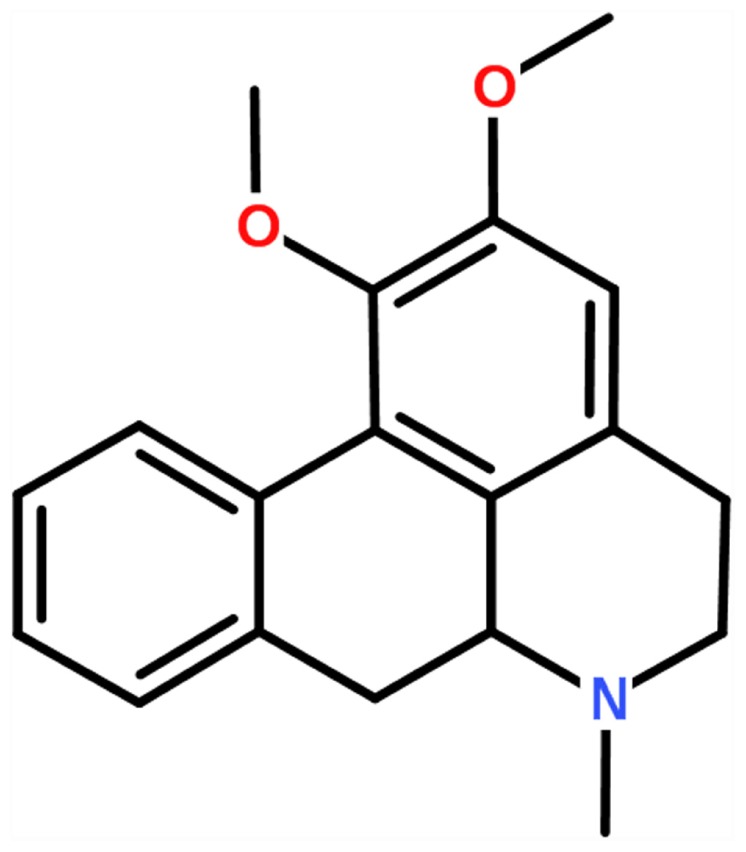
Chemical structure of nuciferine.

Recently, researchers have increasingly focused on natural products for their protective roles in the management and prevention of oxidative stress or inflammatory-related diseases and because they have fewer side effects (Menghini et al., 2016; Ferrante et al., 2017; Locatelli et al., 2017). Nuciferine (C_19_H_21_NO_2_, the chemical structure is shown in **Figure 1**) is a main bioactive component obtained from the lotus leaf and has extensive pharmacological functions, such as anti-cancer, anti-inflammatory, reduced body weight and antioxidant actions (Liu et al., 2014, 2015; Zhang et al., 2015; Qi et al., 2016; Wang et al., 2016). Macrophages are critical components in the connection between innate immunity and metabolism and tissue balance (A-González and Castrillo, 2011). Although the anti-inflammatory activity of nuciferine has been reported, there is no evidence that has elucidated the effects of nuciferine on LPS-induced ALI in mice and RAW264.7 cells.

In the present study, we aimed to explore the anti-inflammatory effects of nuciferine on pro-inflammatory mediator production in LPS-induced ALI in mice and RAW264.7 cells. Additionally, the molecular mechanisms involved were also explored.

## Materials and Methods

### Reagents

Nuciferine (purity > 98%) was obtained from the National Institute for the Control of Pharmaceutical and Biological Products (China). RAW264.7 cells were obtained from the American Type Culture Collection (United States). LPS was obtained from Sigma (United States). IL-1β, IL-6, IL-10, and TNF-α ELISA kits were obtained from BioLegend (United States). Phospho-NF-κB p65 (Ser536) (93H1) Rabbit mAb, NF-κB p65 (D14E12) XP^®^ Rabbit mAb, Phospho-IκBα (Ser32) (14D4) Rabbit mAb, IκBα (L35A5) Mouse mAb, and TLR4, β-actin were obtained from Cell Signaling Technology (Beverly, United States).

### Experimental Processing

Fifty BALB/c mice (8 weeks old) were purchased from the Experimental Animal Center of Wuhan University (China). Mice were exposed to 12-h normal illumination and provided free food intake at room temperature and 65% humidity for 1 week before the experiments. This study was conducted in accordance with the prescribed experimental animal guidelines. All of the agreements were responsible for the Hubei Province experimental animal research center and Huazhong Agricultural University ethics committee (No. HZAUMO-2015-12).

Lipopolysaccharide was dissolved in phosphate buffered saline (PBS). Mice were randomly grouped as follows (each group of 10 mice): control group (PBS), LPS group (0.5 mg/kg), nuciferine (10 and 20 mg/kg)+LPS groups, and nuciferine (10 mg/kg) group. Establishment of the LPS stimulated ALI mouse model was based on a previous literature description (Lv et al., 2015). In brief, intranasal perfusion of LPS to induce ALI in mice. After 24 h of LPS stimulation, an intraperitoneal injection with nuciferine (10 and 20 mg/kg) was performed three times at 6, 12, and 18 h in the nuciferine groups. The same treatment in the control group was performed with PBS. Next, the mice were anesthetized with pentobarbital sodium and executed. Finally, the lung tissues were harvested and preserved in -80°C.

### Histopathological Examination

Ten percent formalin was used to fix lung tissue for 24 h. Next, tissues were embedded in paraffin, sliced, and hematoxylin-eosin stained. Finally, light microscopy was performed to evaluate the pathological changes in lung morphology.

### Lung Wet/Dry (W/D) Ratio

The wet lung tissue from mice was separated and its weight was recorded using a precision electronic balance (SHIMADZU AUY120, Japan). Next, the lung tissue was placed in a constant temperature blower dryer at 60°C to dry for 72 h. The dry weight of the lung tissue was measured again. To estimate pulmonary edema, the lung W/D ratio was calculated as previously described (Wu et al., 2016a).

### Preparation of Bronchoalveolar Lavage Fluid (BALF)

Bronchoalveolar lavage fluid was used to prepare endotracheal intubation and rinsed with sterile physiological saline three times according to the literature description (Abdelmageed et al., 2015). BALF solution was centrifuged at 3000 rpm for 15 min, and the supernatants were later collected and preserved in -80°C until analysis.

### Myeloperoxidase (MPO) Assay

The lung tissues from mice were collected, 100 mg tissues were homogenated with PBS (w/v = 1:19), and the tissue homogenate was subsequently centrifuged at 12000 rpm for 10 min. The supernatant was later collected. Detection of MPO activity in the supernatant was performed with a kit from the Nanjing Biological Co., Ltd., in accordance with the manufacturer’s instructions. The absorbance was measured with spectrophotometry at 460 nm.

### Cell Cultivation and Processing

RAW264.7 macrophage cells were cultured in high glucose DMEM medium supplemented with 10% FBS at 37°C in five percent CO_2_ incubator. Cells were pretreated with nuciferine (10 and 20 μg/mL) for 1 h before being challenged with LPS (0.5 μg/mL) for 6 h. Cells without any treatment acted as the control group.

### Cell Viability Assay

To determine the cytotoxicity stimulated by nuciferine, MTT experiments were carried out in accordance with the manufacturer’s instructions. The cells (1 × 10^5^/mL) were plated in 96-well plates and cultured for 3 h in a 37°C incubator. Subsequently, the cells were treated with nuciferine (10 and 20 μg/mL) for 24 h. MTT (5 mg/mL) was added to the 96-well plate for 4 h, the supernatant was removed, and then one hundred microliters of DMSO was added. The absorbance value was measured at 570 nm with an ELISA Reader.

### Analysis of Cytokines

The effects of nuciferine on LPS induced cytokine secretion were detected in cells and tissues. Tissues were homogenized with pre-cooled PBS, then centrifuged for 15 min at 12,000 rpm. Next, the supernatants were harvested. The supernatants of cells were also collected. The levels of TNF-α, IL-6, IL-1β, and IL-10 in all supernatants were determined with ELISA kits in accordance with the producer’s instructions. The absorbance value was measured at 450 nm with an ELISA Reader.

### qRT-PCR Assay

The total RNA of lung tissues and cells was extracted by Trizol agent. Determination of the purity of RNA was performed at 260/280 nm, and then reverse transcribed into cDNA. Primers used for detecting the expression of mRNA are displayed in **Table 1**. PCR reaction conditions were as follows: 95°C for 10 min, 40 cycles of 95°C for 15 s, 60°C for 60 s, and 72°C for 60 s. The relative expression levels of target genes were standardized with the GAPDH gene using the 2^-ΔΔ*C*_t_^ method.

**Table 1:** Sequence of primers used for quantitative real-time PCR.

Name	Sequence (5′→3′):forward and reverse	GenBank accession Number	Product size (bp)
TNF-α	CTTCTCATTCCTGCTTGTG	NM_013693.3	198
	ACTTGGTGGTTTGCTACG		
IL-6	GGCGGATCGGATGTTGTGAT	NM_031168.1	199
	GGACCCCAGACAATCGGTTG		
IL-10	TGGGTTGCCAAGCCTTATCG	NM_010548.2	118
	TTCAGCTTCTCACCCAGGGA		
IL-1β	CCTGGGCTGTCCTGATGAGAGTCCA	NM_008361.4	131
	CGGGAAAGACACAGGTA		
GAPDH	CAATGTGTCCGTCGTGGATCTGTCC	NM_001289726.1	124
	TCAGTGTAGCCCAAGATG		


### Immunoblotting Detection

Tissues and RAW264.7 cells were lysed with lysate of RIPA adding phosphatase inhibitor and centrifuged at 12,000 rpm for 10 min. The protein concentration was detected using a BCA kit. Next, protein was separated by 10% SDS-PAGE and transported to PVDF membranes. It was sealed with 5% skimmed milk powder for 2 h and the protein was hybridized with the primary antibody at 4°C for 12 h. Subsequently, membranes were incubated with secondary antibodies containing horseradish peroxidase at 25°C for 1 h. The chemiluminescence signal was imaged with the ECL Plus Western Blotting Detection System.

### Small Interfering RNA (siRNA) Transfection

TLR4-siRNA, and negative control siRNA were obtained from GenePharma Co., Ltd. (Shanghai, China). For TLR4-siRNAtransfection, RAW264.7 cells (1 × 10^5^ cells mL^-1^) were grown in six well culture-plates and allowed to reach approximately 60% confluence. Transfection was carried out in Opti-MEM medium with negative control siRNA (NC-siRNA), TLR4-*siRNA* through Lipofectamine^TM^ 2000 in accordance with the producer’s protocol. After 6 h, pretreatment with nuciferine (20 μg/mL) was performed for 1 h, LPS (1 μg/mL) was added for 6 h in the treatment group. Finally, cells were cleaved for further analysis.

### Immunofluorescence Staining

RAW264.7 macrophage cells (1 × 10^4^ mL^-1^) were passaged into 12-well culture plates. An immune fluorescence experiment was carried out after RAW264.7 cells were processed as indicated. RAW264.7 cell slices were incubated with TLR4 antibodies at 4°C for 12 h, and incubated with secondary antibodies in the dark for 2 h at room temperature. Subsequently, the nucleus was counterstained with DAPI and visualized under a fluorescence microscope (magnification 400×). The fluorescence values were processed by Image-Pro software to quantified the IOD and then averaged IOD of each section.

### Statistics Analysis

The SPSS 16.0 software was used to analyze the data. The results were presented as the mean ± SEM. The comparisons between the groups were conducted by an ANOVA followed by Student’s *t*-test. The *p*-values less than 0.05 were considered to be significant.

## Results

### Effects of Nuciferine on LPS-Stimulated Lung Injury

**FIGURE 2:**
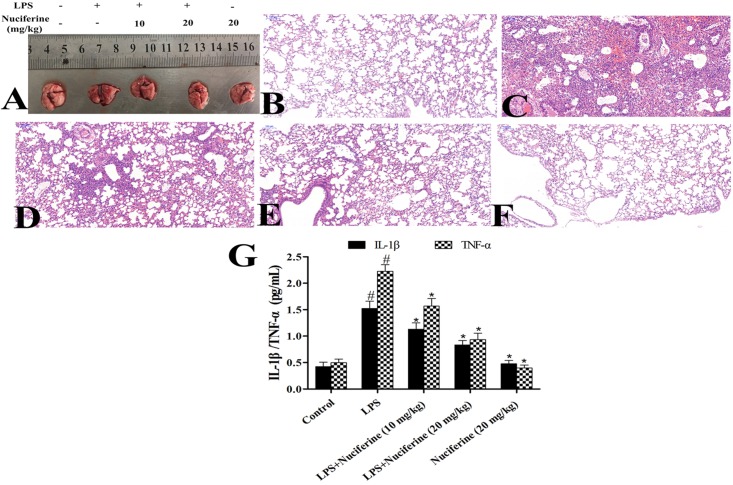
Effects of nuciferine on lipopolysaccharide (LPS)-induced lung injury. **(A)** Morphology of the lung. **(B)** Control group. **(C)** LPS group. **(D,E)** LPS + nuciferine (10 and 20 mg/kg) groups. **(F)** Nuciferine (20 mg/kg) group. **(G)** Bronchoalveolar lavage fluid was determined with an ELISA kit. Data are represented as the mean ± SEM of three independent experiments. ^#^*p* < 0.05 vs. Control group, ^∗^*p* < 0.05 vs. LPS group.

The severity of LPS-induced ALI was determined by histological analysis and the BALF method. Lung morphology was observed in each group (**Figure 2A**). The results displayed that the structure of the alveolar wall was normal in the control group (**Figure 2B**). However, thickening of the alveolar wall, alveolar hyperemia, and infiltration of inflammatory cells were observed in the LPS group (**Figure 2C**). After treatment with nuciferine, alveolar hyperemia, inflammatory cell infiltration and thickening became greatly improvement (**Figures 2D–F**). Additionally, the inflammatory mediators in the BALF group were measured via an ELISA assay. The results indicated that IL-1β and TNF-α secretions were markedly increased after the LPS challenge, which was decreased under nuciferine treatment, especially at a high concentration (**Figure 2G**).

### Effects of Nuciferine on MPO Activity, Lung W/D Ratio and Cell Vitality

**FIGURE 3:**
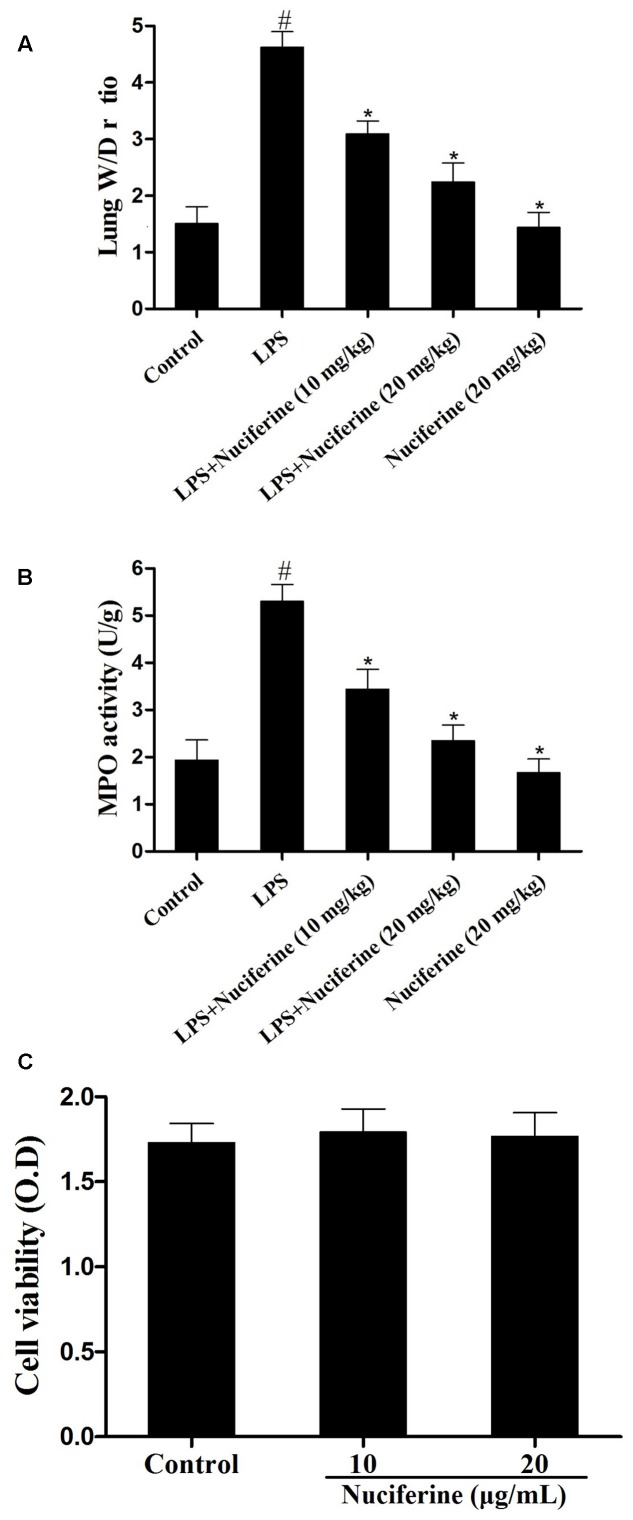
Effects of nuciferine on the lung W/D ratio, MPO activity and cell viability. The lung W/D ratio and MPO activity were determined as described in section “Materials and Methods.” **(A)** Lung W/D ratio. **(B)** MPO activity. **(C)** RAW264.7 cells were cultured with various concentrations of nuciferine (0–20 μg/mL) for 24 h. Cell viability was measured using an MTT assay. Data are represented as the mean ± SEM of three independent experiments. ^#^*p* < 0.05 vs. Control group, ^∗^*p* < 0.05 vs. LPS group.

Myeloperoxidase activity, a marker of neutrophil influx into tissue, is another method to evaluate the phagocytosis of parenchyma infiltration (Krawisz et al., 1984; Wu et al., 2016a). Additionally, it has been reported that pulmonary edema is one of the main characteristics in LPS induced ALI (Qiushi et al., 2015). Thus, the effects of nuciferine on LPS stimulated pulmonary damage were also determined by MPO activity and the lung W/D ratio. As shown in **Figure 3A**, compared with the control group, the lung W/D ratio increased dramatically under LPS stimulation. However, nuciferine treatment could greatly reduce the lung W/D ratio. To further estimate the effects of nuciferine on LPS-stimulated ALI, MPO activity was also assessed. As shown in **Figure 3B**, LPS greatly increased MPO activity, which was significantly decreased with nuciferine treatment. The potential toxicity of nuciferine to RAW264.7 cells was evaluated with an MTT experiment. As displayed in **Figure 3C**, the cell vitality was not susceptible to nuciferine treatment.

### Effects of Nuciferine on Pro-inflammatory Mediator Production

**FIGURE 4:**
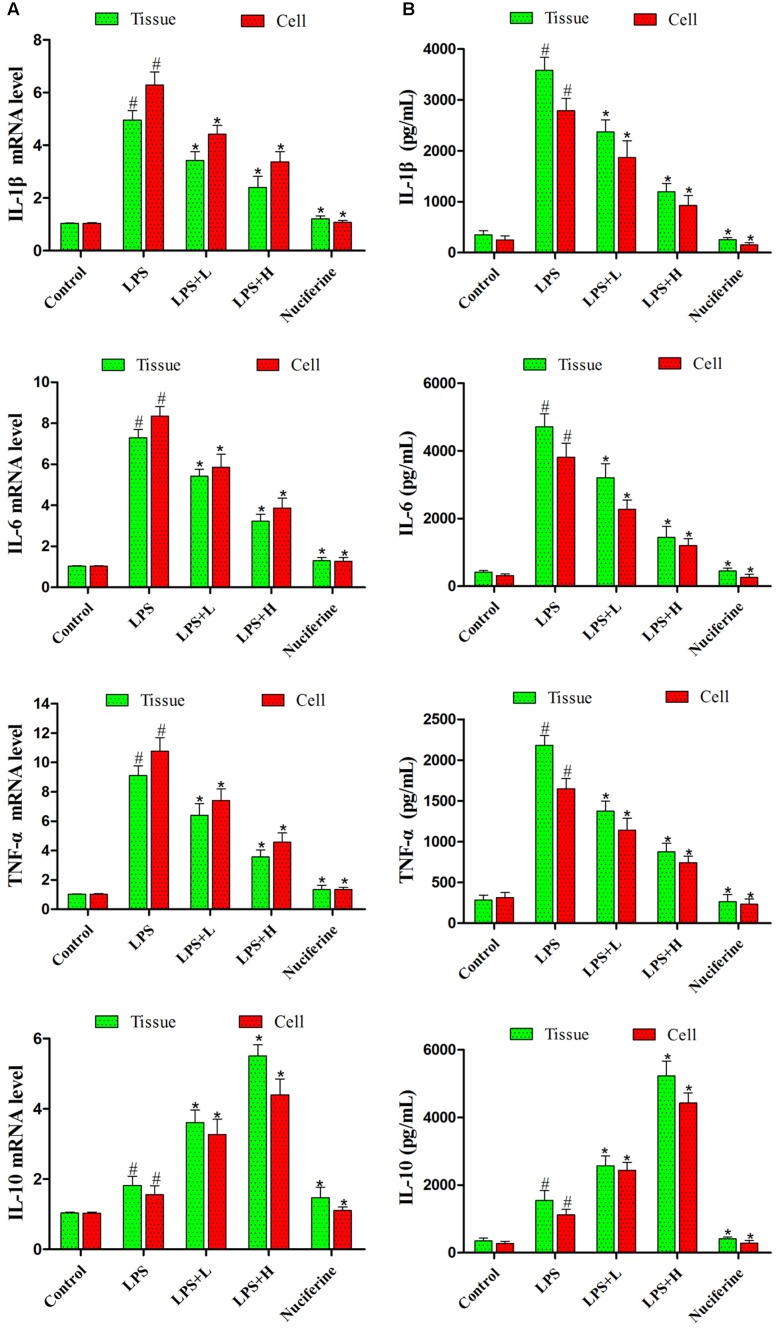
Effects of nuciferine on the secretion of cytokines. **(A)** The level of cytokines TNF-α, IL-6, IL-1β, and IL-10 mRNAs induced by LPS were detected qRT-PCR in tissues and cells. *GAPDH* served as the control. **(B)** The expressions of TNF-α, IL-6, IL-1β, and IL-10 were detected using an ELISA kit in tissues and cells. L and H indicate the tissues treated with 10 and 20 mg/kg nuciferine, respectively, or the cells treated with 10 and 20 μg/mL nuciferine, respectively. Data are represented as the mean ± SEM of three independent experiments. ^#^*p* < 0.05 vs. Control group, ^∗^*p* < 0.05 vs. LPS group.

Pro-inflammatory mediator levels in cells and tissues were detected by qRT-PCR and ELISA assays. qRT-PCR results are displayed in **Figure 4A**. LPS markedly increased the IL-1β, IL-6, and TNF-α mRNA levels. Nuciferine treatment greatly inhibited the TNF-α, IL-6, and IL-1β mRNA levels in tissues and cells, but the expression of IL-10 was increased in tissues and cells. ELISA results suggested that nuciferine treatment inhibited the TNF-α, IL-6, and IL-1β protein expression levels compared to LPS stimulation but increased the IL-10 secretion in tissues and cells (**Figure 4B**).

### Effects of Nuciferineon the Expression of TLR4

**FIGURE 5:**
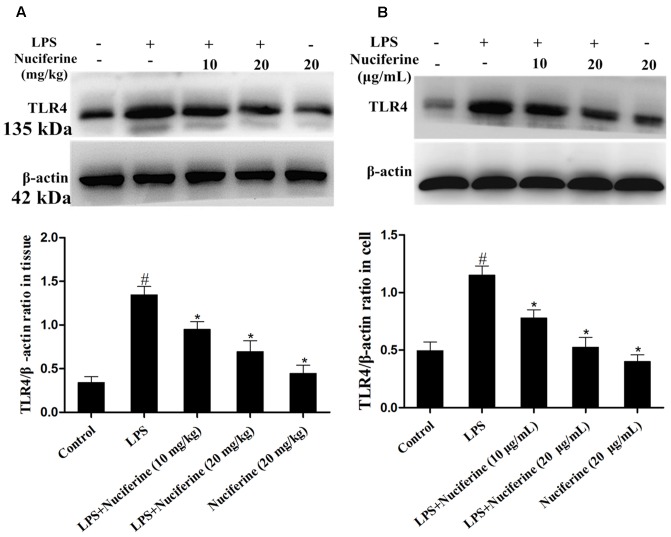
Effects of nuciferine on the expression of TLR4. **(A)** The effects of nuciferine on TLR4 expression was determined by Western blotting in lung tissues. **(B)** The effects of nuciferine on TLR4 expression was determined by Western blotting in RAW264.7 cells. β-actin served as the control. Data are represented as the mean ± SEM of three independent experiments. ^#^*p* < 0.05 vs. Control group, ^∗^*p* < 0.05 vs. LPS group.

TLR4 is identified as a signaling receptor for LPS and is thought to have a role in innate recognition of bacteria (Akashi et al., 2000). The effect of nuciferine on TLR4 expression was detected with an immunoblot assay in tissues and cells. As displayed in **Figure 5**, TLR4 expression was increased by the LPS challenge. Nuciferine treatment obviously suppressed LPS-stimulated TLR4 expression in lung tissues and cells.

### Effects of Nuciferine on Activation of the NF-κB Pathway

**FIGURE 6:**
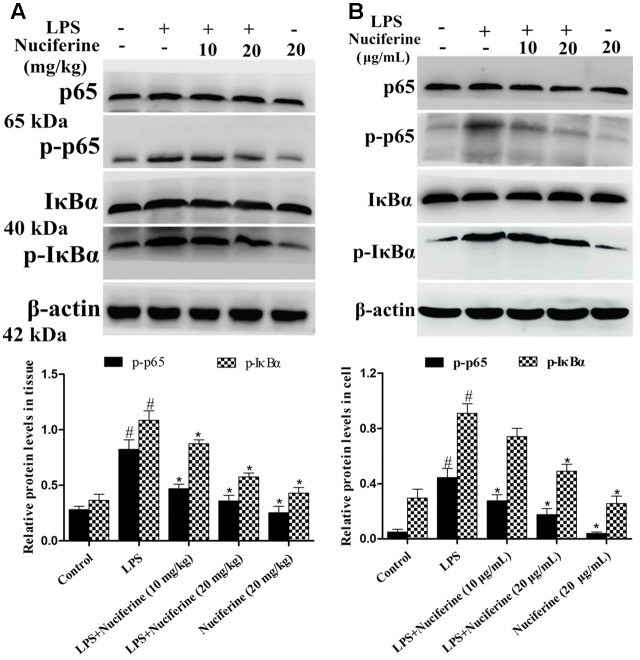
Effects of nuciferine on NF-κB pathway activation. **(A)** Expression of IκBα and p65 proteins in LPS-induced acute lung injury. **(B)** Expression of IκBα and p65 proteins in LPS-stimulated RAW264.7 cells. β-actin served as the control. These antibodies were both obtained from the Cell Signaling Technology [Phospho-NF-κB p65 (Ser536) (93H1) Rabbit mAb, NF-κB p65 (D14E12) XP^®^ Rabbit mAb, Phospho-IκBα (Ser32) (14D4) Rabbit mAb, IκBα (L35A5) Mouse mAb]. Data are represented as the mean ± SEM of three independent experiments. ^#^*p* < 0.05 vs. Control group, ^∗^*p* < 0.05 vs. LPS group.

The NF-κB pathway exerts a vital role in the inflammatory processes of LPS stimulation (Yang et al., 2016). We measured the effect of nuciferine on the LPS-stimulated NF-κB pathway activation in lung tissues and cells using a Western blot method. As shown in **Figure 6A**, the phosphorylation of p65 and IκBα proteins was clearly increased by the LPS challenge. In contrast, the phosphorylation of p65 and IκBα proteins were markedly decreased by the nuciferine treatment. The phosphorylation of p65 and IκBα proteins were greatly increased by the LPS challenge, which was reduced by nuciferine treatment in RAW264.7 cells (**Figure 6B**).

### Effects of TLR4-siRNA Transfection on LPS-Induced Inflammatory Responses

**FIGURE 7:**
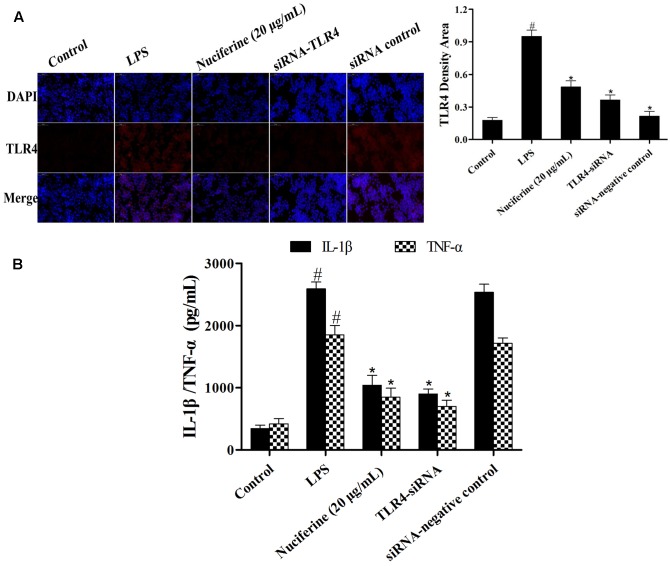
Effects of TLR4-siRNA transfection on LPS-induced inflammatory responses. **(A)** RAW264.7 cells were subjected to transfection with either control siRNA or TLR4 siRNA. The immunofluorescence technique was performed to determine TLR4 expression. Quantification of TLR4 positive density in each group. **(B)** After TLR4 interference, the LPS induced the production of pro-inflammatory cytokines IL-1β and TNF-α, which were measured using an ELISA kit. Data are represented as the mean ± SEM of three independent experiments. ^#^*p* < 0.05 vs. Control group, ^∗^*p* < 0.05 vs. LPS group.

To further confirm the anti-inflammatory mechanism of nuciferine is through the TLR4-mediated pathway, specific interference RNA (TLR4-siRNA) transfection was performed in RAW264.7 cells. RAW264.7 cells were transfected with control siRNA or TLR4 siRNA. The immunofluorescence technique was employed to detect TLR4 expression, and the results indicated that TLR4 expression was markedly decreased by TLR4-siRNA and nuciferine treatment (**Figure 7A**). After TLR4 interference, LPS induced the secretion of pro-inflammatory cytokines, which were measured using an ELISA kit. The results suggested the IL-1β and TNF-α secretions were clearly reduced by TLR4-siRNA and nuciferine treatment (**Figure 7B**). These results suggested that nuciferine inhibited the inflammatory response via the TLR4-mediated NF-κB signaling pathway.

## Discussion

Acute lung injury is characterized by activated neutrophils entering the pulmonary vasculature and alveoli, causing local lung or systemic inflammatory responses (Aulakh et al., 2014). LPS, a part of the cell wall of a Gram-negative bacterium, is often used to induce ALI (Agorreta et al., 2003; Alba-Loureiro et al., 2004; Wu et al., 2016a). It has been shown that nuciferine has several pharmacological characteristics, such as the anti-inflammatory activity (Wang et al., 2016). In this study, we observed that LPS induced inflammatory cell infiltration and alveolar congestion, which was significantly relieved by nuciferine treatment. The results of an *in vivo* study demonstrated that nuciferine relieves inflammatory injury in LPS-induced ALI.

Although inflammation is beneficial to the host’s immune defense, excessive inflammatory reactions cause damage (Kuriakose et al., 2014). Following the innate immune system activation, strong pro-inflammatory signals are generated, which not only plays important role in the stable balance of the immune system but is also important for protecting the host from the harmful effects of most inflammation and subsequent tissue repair (Cohen, 2002; Netea et al., 2004). RAW264.7 cell is not only a good anti-inflammatory screening model *in vitro* but is also frequently used for investigating the mechanism involved (Duan et al., 2010; Hu et al., 2017). It has been reported that inflammatory mediators increase dramatically during the LPS-induced inflammatory process (Agrawal et al., 2016). We observed that the secretions of these pro-inflammatory cytokines were reduced by nuciferine. Moreover, NF-κB served as a major regulator of immunological and inflammatory reactions (Ando et al., 2010). This protein regulates the transcription of various inflammatory cytokines with products that are involved in LPS-induced inflammation (An et al., 2002; Ma et al., 2012). The results showed that nuciferine administration suppressed phosphorylated p65 and IκBα in LPS stimulated ALI and cells.

Toll like receptor signaling is essential for immunity to various intracellular pathogens (Sasindran and Torrelles, 2011). Most of the studies indicated that LPS-regulated activation of TLR4 culminates in NF-κB transcriptional activity and pro-inflammatory mediator secretion (Abreu et al., 2001; Ando et al., 2002; Wu et al., 2016b). Thus, we hypothesized that the anti-inflammatory effect of nuciferine was achieved by suppressing activation of the TLR4-mediated NF-κB pathway. The present data showed that TLR4 expression was increased in the LPS challenge, but it was decreased in nuciferine administration. To further confirm the anti-inflammatory mechanism of nuciferine was via TLR4-mediated pathway, TLR4 was knocked down. When TLR4 was silenced, we observed that LPS induced pro-inflammatory cytokine secretion was significantly decreased by TLR4-siRNA or nuciferine treatment in LPS-induced RAW264.7 cells. Subsequently, the expression of pro-inflammatory mediators activated by NF-κB was determined in TLR4-siRNA interfering cells. The above results indicated nuciferine suppressed NF-κB activation through regulation of the TLR4 pathway.

Taken together, the results of this study demonstrated the pro-inflammatory mediators IL-1β, IL-6, and TNF-α expressions were inhibited by nuciferine administration. Moreover, the therapeutic effects of nuciferine on LPS-stimulated ALI and cells may act by suppressing the expression of TLR4 and its downstream mediated NF-κB signaling pathway. Accordingly, nuciferine might be an effective drug in preventing and treating LPS induced ALI.

## Ethics Statement

All experiments involving live mice were performed according to the stipulated rules for the experimental usage of laboratory animals. All protocols were followed by the Laboratory Animal Research Center of Hubei Province and the ethics committee of Huazhong Agricultural University (Permit number: HZAUMO-2015-12).

## Author Contributions

HW, and GD contributed to the conception and design of the study. HW, KJ, SG, and JY performed assays and furnished the lab study. YY and GZ performed data collection. HW, KJ, and GD conducted data analysis. HW and CQ drafted the manuscript. HW, KJ, and GD revised the paper. The authors have read and approved the final manuscript.

## Conflict of Interest Statement

The authors declare that the research was conducted in the absence of any commercial or financial relationships that could be construed as a potential conflict of interest. The reviewers LB, MP and handling Editor declared their shared affiliation.
